# Nurse-led social entrepreneurship as a career

**DOI:** 10.4102/hsag.v30i0.2700

**Published:** 2025-04-03

**Authors:** Carolina S. Meintjies, Jeanette E. Maritz

**Affiliations:** 1 Department of Health Studies, School of Social Sciences, University of South Africa, Pretoria, South Africa

**Keywords:** nurse-led, social entrepreneur, career, nursing, education, collaboration, sustainability, ventures, social enterprise

## Abstract

**Background:**

The transition from nursing to entrepreneurship introduces unique challenges and opportunities for nurses. This transition requires nurses to acquire knowledge of the complexities of business management, financial sustainability and healthcare regulations while maintaining a commitment to patient care and social impact.

**Aim:**

The aim of the study was to understand nurses’ experience of nurse-led social entrepreneurship as a career.

**Setting:**

The research context was Gauteng, South Africa, and the study focused on nurses who had made the career transition to social entrepreneurship.

**Methods:**

A qualitative research design was employed for the study, with in-depth interviews being conducted with 12 nurse-led social entrepreneurs. Thematic analysis was used to identify key themes related to the challenges and facilitators of nurse-led social entrepreneurship.

**Results:**

Two principal themes emerged. The first concerns gaps in business acumen, including orientation, experience, financing, marketing, as well as navigating healthcare, community, legislation and technology. Psychological issues such as fear and jealousy were noted. The second highlights support systems’ role in overcoming barriers and fostering growth, advocating stronger support and networking for nurse social entrepreneurs.

**Conclusion:**

Nurse-led social entrepreneurs demonstrate resilience in making the transition from clinical roles to business leadership, driven by a passion for healthcare and a desire for positive social change. The availability of support and mentorship significantly influences success in this endeavour.

**Contribution:**

The study contributes insights into the difficulties faced by nurse-led social entrepreneurs and underscores the significance of support systems in facilitating successful entrepreneurial ventures.

## Introduction

Nurse-led social entrepreneurship combines clinical expertise with business strategies to create and sustain social value, particularly in underserved areas where traditional healthcare services may be lacking or inaccessible (Van Ster Ngoie 2024:16–18). This form of entrepreneurship aims to deal with pressing social concerns through innovative solutions, enabling nurses to extend their impact beyond conventional healthcare settings and resolve societal problems effectively (Kabir 2019:215). While historical figures such as Florence Nightingale and Professor Charlotte Searle demonstrated the impact of integrating nursing with social entrepreneurial initiatives (Unisa 2021), Cleopatra Van Ster Ngoie, a local nurse entrepreneur in the Western Cape, South Africa, exemplifies these characteristics in modern-day nurse-led social entrepreneurship (Van Ster Ngoie 2024:5).

Nurses engaging in social entrepreneurship are establishing ventures such as private practices, home healthcare services, wellness clinics and consulting firms and as part of their businesses develop medical products, offer direct patient care, engage in advocacy and offer education and training (Vannucci & Weinstein 2017:57). These enterprises provide significant opportunities for nurses, including independent practices, skilled nursing facilities and consultancy agencies, contributing to healthcare improvement and redefining professional identity (Jakobsen, Qvistgaard, Trettin & Rothmann 2021:4142). Innovation ecosystems such as nurse-led hackathons, incubators and accelerators support the development and scaling of healthcare solutions, fostering a culture of innovation and collaboration (Thepna, Cochrane & Salmo 2023:3183). These multifaceted roles enable nurses to implement comprehensive and innovative solutions to healthcare problems (Van Ster Ngoie 2024:116–117).

However, the path of nurse-led social entrepreneurship is not without obstacles. One significant challenge that nurse-led entrepreneurs face is the assumption that prosocial motives (ventures and initiatives driven by altruistic motives) are essential for achieving positive social outcomes (Kibler et al. 2019:608). Bacq and Lumpkin (2021) offers a different view, arguing that the coronavirus disease 2019 (COVID-19) pandemic demonstrated how businesses could achieve substantial social impacts regardless of their initial motives. This perspective challenges the traditional view of social entrepreneurship, suggesting that nurse-led ventures must demonstrate their impact through tangible outcomes rather than relying solely on prosocial narratives. The need for demonstrable impact aligns with Jakobsen et al. (2021:4143) observation that the expectation of inherent prosocial motives in nursing can limit the acceptance and success of nurse entrepreneurs.

The rapid deployment of solutions during crises like the COVID-19 pandemic presents another significant challenge. Bacq and Lumpkin (2021:286) makes the observation that crises necessitate swift innovation and adaptation, which can be resource-intensive and require quick decision-making. Neergård (2020:1248) agrees, noting that bureaucratic and resource constraints within the healthcare system can impede nurses’ ability to respond effectively. However, Thepna et al. (2023:3184) emphasise that nurses’ inherent flexibility and problem-solving skills could be leveraged to overcome these obstacles, suggesting that while resource constraints are a hurdle, they also allow nurse entrepreneurs to showcase their adaptability and innovation.

Nurse social entrepreneurs often encounter cultural and gender barriers within the nursing profession. Jakobsen et al. (2021:4144) highlight the stereotyped view of nursing as a profession focused solely on patient care, often to the exclusion of entrepreneurial activities. Gender norms and expectations present additional obstacles for nurse entrepreneurs.

Systemic deficits in education and institutional support further compound these cultural and gender barriers. Research by Dzomonda and Fatoki (2019:8) highlights the shortcomings of South African higher education institutions in fostering entrepreneurship, particularly in practical and financial support domains. Thepna et al. (2023:3184) conclude that the lack of entrepreneurial content in nursing education further reinforces these stereotypes. The education gap is significant, and many nursing schools need more business management, financial planning and marketing coursework, as knowledge of these areas is essential for entrepreneurial success. Nwodoh, Chamaka and Nneka (2021:8) and Roslan et al. (2020:9) also identify significant gaps in nurses’ entrepreneurial education and support systems, pointing out a lack of legal and financial knowledge and inadequate curriculum design in higher education. The lack of adequate entrepreneurial education directly impedes nurses’ ability to navigate regulatory and policy landscapes.

The level of support and resources available to nurse entrepreneurs significantly influences their success. Neergård (2020:1248) discusses the way in which limited resources and bureaucratic difficulties within healthcare systems can hinder innovation, and Jakobsen et al. (2021:4144) add that the absence of institutional support can dissuade nurses from pursuing entrepreneurial ventures. These challenges are compounded by systemic issues, such as regulatory frameworks and societal perceptions of nursing, which often do not align with or support entrepreneurial activities (Radebe 2019:68).

Nevertheless, significant opportunities do exist for nurse social entrepreneurs. The global healthcare industry continues to grow (BDO Global 2023), presenting significant opportunities for nurse entrepreneurs to develop and expand healthcare services.

The study reported on in the present article delved into the unique intersection of nursing and social entrepreneurship, where the intrinsic values of care and social improvement meet entrepreneurial action. The aim was to fill this knowledge gap by achieving an understanding of nurses’ experiences as social entrepreneurs. Through the documentation of these experiences, the research contributes to a greater understanding of the intersection between nursing and social entrepreneurship, offering insights with the potential to inform policy, education and practice supporting nurse-led social entrepreneurial endeavours. This exploration is crucial for developing targeted interventions able to facilitate the growth of social entrepreneurship within nursing, thereby amplifying its impact on societal health and well-being.

## Research methods and design

### Study design

A qualitative research design and inductive reasoning were used for the study (Creswell & Creswell 2023:30). Qualitative designs serve as both a methodology and a paradigm. As a methodology, qualitative designs focus on exploring phenomena in their natural settings, using data collection methods such as interviews, observations and document analysis to gather rich, detailed insights. As a paradigm, qualitative designs embody a worldview that values subjective experiences and the meaning individuals ascribe to their experiences, emphasising the importance of context and the co-construction of knowledge by researchers and participants (Polit & Beck 2022:164). A qualitative design aligns with the social constructivist perspective, emphasising the role of social and cultural contexts in shaping individual experiences and outcomes.

Social constructivism posits that knowledge and meaning are constructed through social interactions and cultural contexts (Lincoln & Guba 2013:45). By leveraging social constructivism, the researchers acknowledged the importance of personal and cultural contexts in shaping experiences and outcomes. This perspective permitted a deeper understanding of how nurses’ personal backgrounds, societal influences and cultural norms influenced their professional experiences and practices. Viewing the topic through this lens, the researchers acknowledged that health beliefs and practices are not merely individual choices but are instead influenced by the larger social environment; this, then, made it possible to consider the complex and multifaceted nature of healthcare provision and nursing practice.

### Setting

The study aimed to answer the research question, ‘*What are nurses’ experiences of nurse-led social entrepreneurship as a career*?’ In South Africa’s public healthcare system, nurses make up 56% of all healthcare workers (Rispel 2020:3), with KwaZulu-Natal having the largest population of nurses (26.4%), followed by Gauteng (23.0%) (Matseke 2023:5). All practising nurses in South Africa need to be registered with the South African Nursing Council (SANC). This regulatory body ensures that all nurses meet the required education and practice standards to safeguard public health and safety (SANC 2020).

The study was conducted in Gauteng due to the diverse healthcare environments in which nurses have made the transition to social entrepreneurship in the province. These nurses operated in various settings, including private nursing schools, clinics, non-governmental organisations (NGOs), community-based health services and private practices. This diversity in settings was crucial for capturing a range of experiences and understanding how different environments influence nurse-led social entrepreneurship.

This geographic focus on Gauteng may, however, have introduced certain biases or limitations. Gauteng, a highly urbanised province with a well-developed healthcare infrastructure, may only partially represent the experiences of nurse social entrepreneurs in more rural or under-resourced settings in South Africa. An urban bias (Eckert, Ganapati & Walsh 2020:np) such as this might not have taken account of the unique challenges and opportunities experienced by nurses in other provinces with different healthcare dynamics.

In addition, Gauteng’s economic and social conditions are distinct from those in other regions, potentially leading to an over-representation of issues relevant to urban settings while under-representing rural healthcare issues. As a result, the findings might not be entirely generalisable to the broader population of nurse-led social entrepreneurs across the country.

### Study population and sampling

The study population consisted of nurses who owned private businesses, such as those who opened schools for nursing training, wound care clinics, primary health care clinics, NGOs, counselling services or consultancies. Participants were selected based on lists from a number of organisations where professional nurses and entrepreneurs are registered, including the Health and Welfare Sector Education and Training Authority (HWSETA), the Quality Council for Trades and Occupations (QCTO), the Society of Nurse Practitioners (SNP) and the Board of Healthcare Funders (BHF). Not all nurse entrepreneurs are registered with the HWSETA, QCTO or BHF, and it was therefore not possible to calculate the exact population size.

Purposive and snowball sampling were employed for the study to ensure a comprehensive and information-rich sample relevant to the research questions. Purposive sampling (Creswell & Creswell 2023:198) was chosen to deliberately select participants with specific characteristics or experiences pertinent to nurse-led social entrepreneurship. This method focuses on individuals able to provide deep insights into the phenomenon under study, ensuring rich and relevant data.

Snowball sampling was used to complement purposive sampling. Snowball sampling, as described by Kennedy-Shaffer, Qiu and Hanage (2021:1918), involves having existing study participants recruit future participants from among their acquaintances. This technique is particularly effective for accessing hard-to-reach populations or individuals with rare traits who may not be easily identified by means of traditional methods.

The integration of purposive and snowball sampling provided a robust approach. Purposive sampling ensured that the initial participants were highly relevant, while snowball sampling expanded the reach to include additional participants with similar characteristics. This complementary approach facilitated the identification of a diverse and comprehensive sample.

The inclusion criteria encompassed:

- Nurses registered with the SANC who self-identified as social entrepreneurs and were involved in private businesses, NGOs, nursing schools, clinics and counselling services, among others, within Gauteng, South Africa.
- An age range of 30 years – 65 years to encompass nurses at various stages of their careers who may have embarked on social entrepreneurship.

The exclusion criteria were:

- Organisations that employ nurses in roles that do not align with the definition of social entrepreneurship, such as running nursing schools or private clinics as employees rather than owners.
- Nurses practising outside the borders of South Africa.
- Individuals not registered with the SANC.

The sampling continued until data saturation (Leavy 2022:90) was reached. Saturation occurs when no new information or insights are gained, indicating sufficient sample size. Saturation was achieved with a sample size of 12 nurses.

### Data collection

Data were collected by means of 12 in-depth online interviews using Microsoft Teams. Online interviews have both advantages and disadvantages (Fan et al. 2023:10). Advantages include reduced costs and logistical difficulties, as they make it easier to include a wider participant pool by overcoming geographical constraints. Furthermore, participants can join from their preferred settings, which may lead to more honest responses. These factors in combination enhance the efficiency and reach of data collection.

However, online interviews also have notable disadvantages. Technical difficulties, such as connectivity problems, can disrupt the flow of interviews. The inability to observe body language and facial expressions limits the interviewer’s capacity to pick up on non-verbal cues, which can reduce contextual understanding. Building rapport with participants is more difficult and potentially affects the depth of responses. Privacy concerns, environmental distractions and accessibility issues, such as unreliable internet connections or the lack of the necessary devices, further complicate the process.

Despite these challenges, online interviews were conducted so as to leverage the advantage of this form of data collection. Allowing participants to participate from their preferred settings potentially yielded more honest responses. Furthermore, online interviews reduced the costs and logistical difficulties associated with face-to-face interactions, reducing geographical constraints so as to include a wider participant pool. The adoption of online interviews achieved greater flexibility and convenience for participants, in that way enhancing the quality and diversity of the data.

Interviews were scheduled at the participants’ convenience, in that way promoting safety during the COVID-19 pandemic, eliminating travel expenses and requiring only an hour of each participant’s time. A week before the interview, participants received an email requesting their acceptance of an invitation to an MS Teams meeting scheduled for a specific time and lasting 60 min. All participants agreed to the MS Teams option, and meeting requests were sent with the consent form. Participants confirmed receipt of the MS Teams invitation. Where necessary, the researcher offered training or a practice session before the interview. Participants were allowed additional time to connect, with telephonic assistance provided until they had successfully logged in to MS Teams. Additionally, the researcher offered to sponsor data through the First National Bank app, using the participants’ phone numbers, up to R100. Participants were free to either accept or decline this offer.

A pilot interview was conducted with a participant who was subsequently included as an interviewee, as no changes to the questions were necessary. Pilot interviews in qualitative research make it possible to refine questions, improve techniques and identify possible problems before the main study. Pilot interviews enhance trustworthiness and allow researchers to practise interviewing skills. Additionally, they reveal necessary modifications to the research instrument and help anticipate problems in participant recruitment and engagement (Hui, Halili, & Razak 2022:1462–1482).

The interviews conducted by the first author focused on exploring the experiences, challenges and achievements of nurses as social entrepreneurs. The two grand tour questions were:

1. *Tell me about your experience as a social entrepreneur?*
2. *What support do you think nurses need when considering social entrepreneurship as a career?*

The first question, ‘Tell me about your experience as a social entrepreneur?’, aimed to capture nurse social entrepreneurs’ diverse experiences and insights. The second question, ‘What support do you think nurses need when considering social entrepreneurship as a career?’ sought to identify the specific needs and resources necessary for nurses making the transition to social entrepreneurship. This approach ensured comprehensive data collection relevant to the objectives of the study. Through the study, the researcher aimed to highlight potential areas for intervention and policy development to support nurses in their entrepreneurial pursuits.

To enrich the data, special attention was paid to verbal and non-verbal cues. Interviews were recorded with participants’ consent, and all data were kept confidential and accessible only to the research team. The second author performed quality checks on all the recorded interviews and transcriptions. Field notes comprised information relevant to the research questions and any unexpected insights that emerged during the interviews (Creswell & Creswell 2023:199).

### Data analysis

The study was conducted to investigate the experiences of nurse led as career social entrepreneurs within the South African healthcare system, with a specific focus on Gauteng. By exploring nurses’ experiences and identifying the support systems required, the researcher sought to provide valuable insights with the potential to inform policy and practice, thereby enhancing nurses’ role in offering innovative solutions to fill healthcare gaps.

Creswell and Creswell (2023:207–208) describe a rigorous process for conducting thematic analysis, which the researcher adhered to. The process incorporated the assistance of a co-coder and oversight from the second author throughout. Initially, the data were organised and prepared for analysis, which included the careful review of transcribed material and the preparation and typing up of observation and field notes.

Atlas.ti software version 23.2.2.27458 was used to assist in the data analysis, and its artificial intelligence feature was employed to validate the codes identified by the researcher (Leavy 2022:19; Saldaña 2021:6). Twelve interview transcripts were imported into Atlas.ti 23 after the project was created; the program was employed to identify and extract 301 quotations from the data, and codes were created from these quotations. Text was collected, and codes were applied. Codes were arranged into groups, for example ‘business’, ‘challenges’, ‘collaboration’, ‘marketing’, ‘mentoring’, ‘motivation’, ‘personal development’, ‘professional development’, ‘psychological well-being’ and ‘support’. Coded segments of text that related to specific codes were retrieved. Memo groups were also created to summarise thoughts for insight during data analysis. The researcher and a co-coder then separately immersed themselves in the data, reviewing all materials to understand their collective significance.

Finalising the thematic structure involved defining and naming the themes, with the researcher striving to clearly articulate the essence of each theme and the relationship to the data. The second author was actively involved throughout the data collection and thematic analysis process, with their contribution including listening to the audio recordings and providing feedback for improvement.

### Measures of trustworthiness

In ensuring the trustworthiness of qualitative research, the adoption of strategies that uphold the validity and integrity of the information obtained is crucial. The study adhered to the guidelines set by Lincoln and Guba (1985) relating to credibility, transferability, dependability, confirmability and authenticity. Each of these measures was operationalised as described below.

The research employed prolonged engagement and persistent observation to establish credibility, allowing an in-depth understanding of the participants’ experiences (Miles, Huberman & Saldaña 2020:1127). The researcher devoted significant time to building trust with participants and conducting follow-up interviews where necessary. Reflexivity was practised, with the researcher maintaining a reflective diary to acknowledge and manage potential biases (Dodgson 2019:1). Triangulation was achieved through the involvement of a co-coder, with the credibility of the findings being ensured through the achievement of consensus in the case of coding discrepancies (Creswell & Creswell 2023:215).

The transferability of the findings was ensured by providing detailed, thick descriptions of the study context, participants and findings (Creswell & Creswell 2023:222; Polit & Beck 2022:285). Detailed descriptions made possible the potential application of findings to other contexts, facilitating comparisons by future researchers or practitioners.

Dependability was achieved through detailed documentation of the research process and the maintenance of consistent data collection methods. An audit trail was established, capturing all decisions, methodologies and data analysis processes to ensure that the findings of the study were stable over time and conditions (Creswell & Creswell 2023:265; Polit & Beck 2022:276–288). The involvement of a co-coder and engagement in reflexive journaling further supported the dependability of the research.

Confirmability was achieved by ensuring that the findings reflected the participants’ views rather than the researcher’s preconceptions. An audit trail of the data collection and analysis processes was meticulously maintained, allowing for the verification of how conclusions were drawn (Creswell & Creswell 2023:19; Polit & Beck 2022:508). Multiple data sources and an unbiased approach to data analysis supported this objective stance.

The authenticity of the study was demonstrated through accurate representation of participants’ experiences, which ensured that the diversity of perspectives was captured and faithfully presented (Polit & Beck 2022:509). Using MS Teams for interviews allowed for the recording of both verbal and non-verbal cues, contributing to a reflection of the participants’ realities.

### Ethical considerations

The University of South Africa’s College of Human Sciences Research Ethics Review Committee granted this study’s ethical approval (Reference no.: 32297963_CREC_CHS_ 2021). Participants were informed about the aims of the study and their rights, including privacy and confidentiality. Informed consent was obtained from all participants, including consent for the recording of interviews. Measures were taken to ensure the anonymity of participants and the sensitive handling of the information shared, which was in line with the British Sociological Association Statement of Ethical Practice (2017:6). To overcome the limitations of online interviews, measures were taken to maintain confidentiality and ensure participant comfort. All participants were informed about the measures taken to secure their data, and interviews were conducted using secure, encrypted platforms to protect the privacy of the conversations. In addition, the co-coder signed a confidentiality agreement.

To address the ethical implications of offering data sponsorship, the researchers ensured informed consent, maintained voluntariness and protected participants’ privacy and confidentiality. All participants were offered the sponsorship equally, and the study received ethical approval for this. In the end, however, no participants took up the offer of data sponsorship.

Participants were assigned unique identification codes, and to ensure anonymity, any identifying information was removed from the transcripts. These steps helped mitigate privacy concerns and encouraged participants to share openly, secure in the knowledge that their information was protected.

## Results

### Demographic details

The study involved a diverse group of 12 participants, comprising 11 women between the ages of 45 years and 76 years, and one 59-year-old male. Half were in their mid-career (between the ages of 35 years and 54 years), and half were approaching or at retirement age (55+ years).

The highest qualifications reported include Clinical Master Nursing Education obtained in Australia, General Nursing, Registered Nursing with a Master of Business, BA Cur Honours and up to a PhD in Nursing. One participant combined a background in nursing with qualifications in law, holding both a degree in law and an LLM in medical law.

The business enterprises operated by the participants were equally diverse, relating to various sectors within the healthcare industry. These ranged from community health training schools and wound care clinics to more specialised offerings such as home-based and palliative care services, healthcare services focusing on wound care, training and development schools, a primary healthcare clinic and a rehabilitation and nursing agency. In addition, one participant was engaged in psychotherapy and business coaching and another operated a microenterprise in entertainment, leadership and mentoring. There was only one male participant, with specialisation in trauma nursing and offering home-based care; this illustrated the predominance of female leadership and entrepreneurship within this group’s healthcare-focused enterprises. Table 1 provides an overview of the demographic data.

**TABLE 1:** Participants’ demographic details.

Participant	Age (years)	Gender	Highest qualification	Business enterprise
P1	65	F	Clinical Master Nursing Education (Australia)	School: Community health training
P2	65	F	General Nurse	Wound care
P3	67	F	Registered Nurse Master of Business	Training and development school
P4	63	F	BA Cur Honours	Healthcare services and wound care
P5	76	F	BA Cur Community and Nursing Education	School: Community health training
P6	52	F	Diploma in Nursing (General, Psychiatric and Community) and Midwife, specialised in Palliative Care	Home-based and palliative care, nursing agency, rehabilitation
P7	48	F	PhD Nursing	Wound care clinic
P8	45	F	Degree in Nursing	Primary healthcare clinic
P9	60	F	D Cur Nursing	Psychotherapist and business coach
P10	57	F	B Cur degree	Microenterprise entertainment, leadership and mentoring
P11	49	F	Diploma in Nursing (General, Psychiatric and Community) and Midwife, Degree in Law and LLM Medical Law	Advocate
P12	59	M	Registered nurse specialised in trauma	Home-based care

P, participant; F, female; M, male; BA Cur, Baccalaureus Curationis; PhD Nursing, Doctor of Philosophy in Nursing; B Cur, Bachelor of Arts Nursing Science; LLM, Masters of Law; D Cur Nursing, Doctor Curationis Nursing.

Table 2 summarises the themes and categories. The codes are described within the categories’ narrative.

**TABLE 2:** Themes and sub-themes

Themes	Sub-themes
1. Navigating Entrepreneurial barriers in social ventures	1.1 Lack of business acumen and operational requirements 1.2 The psychological experience of social entrepreneurs 1.3 Thriving amidst challenges
2. The critical need for support, mentorship and networking	2.1 Value of support, mentorship and networking

### Theme one: Navigating entrepreneurial barriers in social ventures

In making the transition from nursing to entrepreneurship, nurses encounter unique challenges stemming from the need to bridge business knowledge and acumen gaps. This theme delves into the critical areas where nurse entrepreneurs often lack the skills and understanding necessary to launch and sustain their ventures successfully. The theme highlights the essential need for a solid foundation in business principles, including orientation, experience and compliance with tax regulations. Furthermore, the theme explores the complexities of financing and marketing a business, which is pivotal for the survival and growth of any entrepreneurial endeavour.

#### Lack of business acumen and operational requirements

Business practices relating to ethics, responsibility, finances, customers, employees, quality assurance, compliance, legalities, health and safety procedures are necessary to manage a business successfully. Participants highlighted a lack of knowledge and planning related to the commencement of a business venture, as is evident in the following extract:

‘I was not, uhm, actually uhm hmm business orientated. I learned the business by opening this business.’ (P1, 65 years old, F)

Participants pointed out that they needed training in managing a business successfully, which included financial planning, understanding tax compliance and human resource management, as expressed in the extracts below:

‘So, to find out, you know what’s the tax in applications for you as a person or for your business in making sure that you have the relevant training and backup.’ (P9, 60 years old, F)

‘I would definitely say you need some training in human resources yeah I think financial management …’ (P12, 59 years old, M)

Participants used free online business management platforms for guidance to ensure that the correct practices were followed:

‘When it comes to business management, again, in our case, there’s a free online and financial system that we use.’ (P10, 57 years old, F)

Motivation and passion were the driving forces behind commencing a business, but a number of participants reported not having considered the financial aspects necessary for sustainability. The nurse social entrepreneurs were motivated more by the desire to create positive social change rather than profit. However, it is necessary to generate income to sustain the business:

‘Which I have to remind myself continuously, and that’s having enough heart to care, but having enough head mind to run a business, and that is difficult, especially if you come from a nursing background because you want to look after the patient you want to care for them. But you must also make money.’ (P7, 48 years old, F)

Participants explained that finances were not available when they began their ventures. They received no financial support from banks or financial institutions. Furthermore, participants had not anticipated that they might not generate income for several months, as revealed in the quotations below:

‘I called a bank. They told me I must have, um, to buy equipment, I must at least have half a million in my bank account. I need money to make money. They say I must have 500,000.’ (P4, 63 years old, F)

‘And, you know, there were times in the year when you didn’t have income.’ (P9, 60 years old, F)

Some participants closed their businesses when they realised they were not generating an income. A participant explained:

‘So, from there, I decided no, I can’t continue with the nursing school. So, I decided to write books. So, I’ve written two, and I’ve published two books.’ (P3, 67 years old, F)

Along with the need for a comprehensive understanding of finances and business management when planning a social enterprise, effective market analysis and marketing skills were acknowledged as necessary for promoting the social enterprise, as noted by the participants quoted below:

‘But I think what’s important is, um, we, you need to do a thorough market analysis on where you want to do the business so that you can have a full understanding of the needs of the markets. What do they need? Is there a service for what you want to provide? So, a market analysis is very, very important.’ (P4, 63 years old, F)

‘[*M*]arketing is a great deal of it, so we so yeah spend lots of time on marketing also.’ (P12, 59 years old, M)

Delays in the registration and accreditation processes of some businesses caused participants stress. Particularly problematic were organisations such as the Health and Welfare Seta (HWSETA) and the QCTO, which delayed service provision and resulted in income loss, as expressed below:

‘So, I decided that this is the fifth month I’ve been paying rent. There hasn’t been any acknowledgement of receipt of my documentation.’ (P3, 67 years old, F)

‘In that, I tried to get off the ground at QCTO accreditation in training, but with that didn’t work out too well because we had Covid in between, and the QCTO closed down basically.’ (P7, 48 years old, F)

Some participants who provided training had to sign 1-year leasing contracts for premises to accommodate verification visits from the QCTO. They were permitted to offer training only once accreditation was granted, which meant that they earned no income during this lease period. Participants also faced difficulties in securing a suitable venue from which to provide their services, as explained below:

‘And then the fifth thing, it’s difficult to get a place where you are going to conduct business.’ (P5, 76 years old, F)

‘We did have about a three-month period when we had to find the correct site for what we intended to start, and we wanted somewhere private, secluded, not in a shopping centre environment and not in an urban environment, let me say, not between houses and buildings. So, we eventually did find this space and a shipping container that’s in an open space.’ (P10, 57 years old, F)

Along with difficulties relating to accreditation and registration, medical aids often did not reimburse private practices for services rendered to patients without a motivation submitted to the medical aid scheme. They specified which services they would cover, limiting the care provided by the nurse to the patient, as highlighted in the subsequent passages:

‘And you have to write a report every time, and you have to motivate and. Um. It’s getting more and more difficult … What this stock is you going to use some medical aids are very difficult. You have to use what you quoted for. If the wound changes and you want to change, you have to write a new follow-up report … Now we have to send, the first time you see a patient, you have to get authorisation and send photos.’ (P2, 65 years old, F)

‘And medical aids, and I’m not going to name the medical aids, that only funds care at home for three months.’ (P12, 59 years old, M)

The communities that nurse social entrepreneurs served added to the complexities of delivering services. Nurse social entrepreneurs found it impossible to distance themselves from community-related problems and expectations, such as patients requesting free services, as described below:

‘And then I’ve found that I am busy solving the community issues.’ (P1, 65 years old, F)

‘Delivering the service part becomes a challenge in a sense that you don’t really want to tell a patient they must pay because you know you actually thought of as Florence Nightingale. But you still need to make money.’ (P7, 48 years old, F)

Once the nurse social entrepreneurs started their businesses, they discovered that they needed a competitive advantage. Nurses explained that specialised services, such as wound care, were provided by some pharmacies at a lower rate, and that the pharmacies did not employ a wound care specialist for this. This negatively affected nurses who specialised in wound care. Additionally, business entities not accredited for training community health workers outsourced these services to nurse entrepreneurs at reduced rates, as revealed by the following extracts:

‘Everybody can do it and say ok, I’m a wound care specialist, and I’m doing wound care because I’m a pharmacist.’ (P2, 65 years old, F)

‘Let me tell you. It’s because people who have got nothing to do with nursing are the ones who are running the schools. And they employ us. We do their work.’ (P5, 76 years old, F)

Not only did the nurse entrepreneurs face human and resource difficulties, but participants also emphasised the stark contrast between the practical, hands-on nature of nursing and the demands of business management, including the use of technology and document drafting:

‘And I think that was my biggest challenge was technology and drafting documents, writing, reading a lot. It’s not like nursing that is very hands-on and practical it’s very different.’ (P11, 49 years old, F)

‘It’s something that I sort of learned the hard way, and I had to pay people to teach me how to set up a contact with the patient, how to and what type of forms does the patient needs? What does the form need to look like, where if the patient visits the clinic, what do they sign? What do I complete? What is the best way to keep those files?’ (P7, 48 years old, F)

Nurse entrepreneurs’ lack of business acumen and operational knowledge ties in with the broader theme of navigating entrepreneurial barriers. Significant gaps in essential business knowledge and planning hindered their ability to overcome the difficulties associated with launching and sustaining their ventures.

#### Psychological experience of social entrepreneurs

The difficulties faced by nurse entrepreneurs did not only relate to business acumen and operational requirements; they often experienced psychological and emotional turmoil, with fear, professional jealousy and reduced psychological well-being all being obstacles.

A participant had concerns about taking the first step when commencing their planned ventures:

‘The worry about your possible failure? Who will try and steer you away from something that they think that you might not succeed in? … If you are someone that is very reserved and you don’t take, umm, that chances in life because you’re afraid to do it, you’re always going to stay … In my case, specifically the fear of not having enough funds to cover everything … Probably the biggest thing I learned is not to be afraid to take the first step … Also, not to initially think that you will have all the answers, and it’s like climbing stairs. You know, you go up a few steps, and then you have to kind of raise, look around, maybe turn back and then start again. And not being afraid to say that this did not work, let me try an alternative way.’ (P10, 57 years old, F)

The high crime rate in South Africa increased participants’ fear as they made the transition to social entrepreneurship. Some participants experienced hijackings, theft of stock and equipment and personal threats:

‘When I did home visits, I got hijacked, and I’ve lost like everything in the car, everything in the boot, and there was like R30000 worth of stock and a camera and a blood pressure machine and a blood glucose machine, everything in the boot.’ (P7, 48 years old, F)

‘And he was, you know, he told me he had a gun in his car, and he threatened me. I was scared for my children, and I decided to stop the therapy from home, and I closed my practice.’ (P9, 60 years old, F)

Professional jealousy was an obstacle to guidance and mentorship, as competition for business made collaboration difficult. Participants explained:

‘There is a lot of professional jealousy. There is no understanding of what you really do, I think for some people, it is an eye opener.’ (P2, 65 years old, F)

‘There’s a fear that they, that of competition, you know.’ (P6, 52 years old, F)

Balancing work and social life, coupled with stress and anxiety due to financial constraints, took a toll on participants’ psychological well-being. Uncertainty about the success of the business and working alone without backup often exacerbated the emotional strain and proved overwhelming to some participants:

‘It is getting so overwhelming, and I let my practice grow. I, as I said, for 27 years now.’ (P2, 65 years old, F)

‘It’s so stressful.’ (P8, 45 years old, F)

‘That worry about your possible failure.’ (P10, 57 years old, F)

Being anxious all the time led to personal neglect, burnout and stress, as a participant noted:

‘I am quite exhausted, and it started to, umm, have an impact on my mental health, and it started to be traumatic … I had two teenagers, so I think I was already, you know, emotionally quite drained and overwhelmed.’ (P9, 60 years old, F)

A participant cautioned that nurse social entrepreneurs need to be aware of this risk and take preventive actions:

‘So very important that you also put yourself and your own welfare upfront and take care of that before, but otherwise, you become an empty vessel, and I don’t think you can be a container for other people.’ (P9, 60 years old, F)

These experiences tie in with the larger theme of navigating entrepreneurial barriers in social ventures by demonstrating that overcoming psychological and emotional challenges such as fear, professional jealousy and stress is as crucial as acquiring business acumen and satisfying operational requirements for nurse entrepreneurs’ sustained success and well-being.

#### Thriving amidst challenges

Not all experiences were negative. Some participants thrived during the COVID-19 pandemic, while others closed their doors, as described below:

‘You build it, and you’ll have good months and bad months, and you have a very busy month where you see lots of patients, and then you have a month like during Covid.’ (P7, 48 years old, F)

‘Yes, we grew 100%, ok a bad thing is that we really worked hard during Covid, so Covid was good for business, but it also added these challenges because then everything and again everything runs down to a financial challenge, a box of gloves of 100 is now R46 during Covid it was between R200 and R300.’ (P12, 59 years old, M)

These experiences illustrate the larger theme of navigating entrepreneurial barriers in social ventures. Some participants managed to thrive despite the challenges posed by the COVID-19 pandemic, highlighting the resilience and adaptability required to sustain and grow their businesses in fluctuating circumstances.

### Theme two: The critical need for support, mentorship and networking

The journey into nurse-led social entrepreneurship is not one to be undertaken alone. Support, mentorship and networking form a triad of critical resources for nurse entrepreneurs. Participants underscored the collective effort required to ensure the sustainability and impact of ventures in the competitive and complex world of healthcare entrepreneurship.

Participants highlighted that support and guidance from institutions such as Health and Welfare Seta (HWSETA) and professionals could have assisted them during the initial phase of planning their ventures, as highlighted in the extracts below:

‘I needed support from HWSETA, telling me about this product and then telling me the outcome of this product, whether it will be beneficial for me or is beneficial just to uplift the community.’ (P1, 65 years old, F)

‘Support from your doctors, your specialists, because you can, as an individual, you won’t be able to provide the patient with holistic care.’ (P4, 63 years old, F)

Participants viewed peer support as essential for making informed decisions. Some participants joined associations such as the Private Nurse Practitioner Association so as to receive this support, which provided valuable guidance through education in marketing and legislative guidelines:

‘I must say one thing we’ve got is a private nurse practitioner association, and they are excellent.’ (P6, 52 years old, F)

Family support was mentioned during the interviews and was considered crucial for success as a social entrepreneur:

‘It’s usually family and friends who love you dearly. That worry about your possible failure.’ (P10, 57 years old, F)

Participants agreed that mentoring could have provided them with much-needed guidance during the initial stages of starting their social ventures. As detailed below, the participants would have greatly valued guidance from experienced mentors:

‘I needed a mentor when I started this, who is going to tell me the … the uhm the advantages and disadvantages of the business.’ (P1, 65 years old, F)

‘So, you need somebody to mentor you and to guide you, and there is a need for support from other nurses in the same field.’ (P4, 63 years old, F)

‘Some way where you have like-minded people that you can share with.’ (P9, 60 years old, F)

‘There’s always someone who could teach you more. Who can mentor you and improve things.’ (P10, 57 years old, F)

Networking was seen as essential, as it was a source of information about which organisations to approach for support and advice. Unjani Clinic, a non-profit organisation, offers funding and guidance to black female professional nurses looking to open primary healthcare clinics in the community:

‘They actually are the ones that go out there and look for funders that can actually help us start. And then, for the first two years, they support you financially.’ (P8, 45 years old, F)

Networking and collaboration with other professionals were viewed as important. As one of the participants noted:

‘Partnerships it can either be with other nurses, doctors, specialist, where you will refer complicated wounds to. The service providers of the different wound care products because they are very helpful.’ (P4, 63 years old, F)

These experiences underscore the larger theme stating that the journey into nurse-led social entrepreneurship requires a collective effort. Support, mentorship and networking are critical to overcoming obstacles and ensuring the sustainability and impact of ventures in the complex healthcare environment.

## Discussion

Participants’ educational backgrounds were varied and rich, encompassing a broad spectrum of qualifications within the nursing field and beyond. With regard to the age of the nurse entrepreneurs, retirement or retrenchment did not diminish their desire to remain relevant and contribute through their extensive nursing experience; this highlighted the social motivations behind their entrepreneurial ventures. This approach shifts the narrative from merely correcting the shortage of nurses to recognising the invaluable, innovative and continuous contributions made by nurses to their communities and workplaces (MacLeod et al. 2021:9).

Nurses making the transition to entrepreneurship face unique difficulties, particularly in bridging business knowledge and acumen gaps, as nurse entrepreneurs often lack the skills and understanding necessary to launch and sustain their ventures (Neergård 2022:2354). Participants identified a lack of knowledge and planning related to the establishment of a business, including financial planning, tax compliance and human resource management. This aligns with the observation made by Cardon and Arwine (2023:50) that entrepreneurs may feel unsuited to their role, leading to occupational exit or venture failure.

Participants faced significant difficulties in securing initial funding, with banks requiring nurse entrepreneurs to have substantial capital before approving loans. Further, participants had not anticipated the lack of immediate income, leading some to close their businesses or embrace alternative ventures. Delays in business registration and accreditation processes, particularly with organisations such as HWSETA and QCTO, caused significant stress and income loss, reflecting the regulatory and financial problems discussed by Thepna et al. (2023:2). Moreover, the psychological toll exerted on nurse entrepreneurs, including emotional turmoil and stress, is consistent with the findings of De Lisser et al. (2024:2) on the impact of a negative work environment on burnout.

The findings of the study as these related to the impact of crime on entrepreneurial activities align with the work of Churchill et al. (2023), who found that higher community crime rates negatively affect entrepreneurship by reducing social capital. Specifically, crimes against people and property diminish key facets of relational social capital – such as trust, voluntary membership of community bodies, support and cooperation – in that way inhibiting entrepreneurial activities. This underscores the critical role of social capital in mitigating the adverse effects of crime on socio-economic outcomes.

Support, mentorship and networking emerged as crucial resources for nurse entrepreneurs. Participants emphasised the need for a collective effort to ensure sustainability and impact, with support from institutions such as HWSETA and peer associations deemed essential. Family support was also seen as vital for success as a social entrepreneur. Mentorship offered by experienced individuals was considered particularly valuable as a means to navigate initial challenges; this information is borne out by the findings of Van Coller-Peter and Cronjé (2020:55). Networking provided valuable guidance and support, with initiatives such as Unjani Clinic (2023) offering funding and mentorship to black female nurses (Szerb, Kivleniece & Aggarwal 2021:94). Collaboration with other professionals and organisations was seen as beneficial, enhancing the overall support system for nurse entrepreneurs. Lleva et al. (2023:351) mention support networking as essential for support and career development.

The findings underscore the need for targeted educational programmes and supportive financial frameworks to bridge the business knowledge gap among nurse entrepreneurs. Policymakers should integrate business training into nursing curricula and provide continuous professional development opportunities. Streamlining registration and accreditation processes and offering tailored funding solutions, such as grants and low-interest loans, could alleviate financial and bureaucratic burdens. Robust mental health support systems, including access to mental health resources and mentorship programmes, are essential to help nurse entrepreneurs overcome the psychological obstacles they face. Support for initiatives such as Unjani Clinic (2023) and the fostering of partnerships between healthcare institutions and business development organisations have the potential to create a supportive ecosystem that enhances the success and sustainability of nurse-led social enterprises.

## Conclusion

The study explored the role of social entrepreneurship in nursing, highlighting the importance of business skills, intrinsic motivation in the form of a passion for healthcare, mentorship, networking and support systems. Although the research offers insights into the entrepreneurial path, limitations included a narrow sample, and it is suggested that future studies should perhaps explore broader experiences in a variety of healthcare settings. Future research could examine the long-term effects of nurse-led enterprises on community health, especially in underserved areas, and the potential for digital technology to enhance these ventures.
